# Optimization strategies for voriconazole dosing in pediatric populations: integrating therapeutic indications and age-stratified pharmacokinetics

**DOI:** 10.1128/aac.01169-25

**Published:** 2026-02-13

**Authors:** Yidan Zhu, Jinxia Zhao, Jiali Chen, Yiru Gong, Nan Guo, Yinyu Zhao, Yuanyuan Li, Jialu Bian, Xuchen Song, Yuxuan Yao, Xingxian Luo, Xiaohong Zhang, Lin Huang

**Affiliations:** 1 Department of Pharmacy, Peking University People’s Hospital, Beijing, China; 2 School of Pharmaceutical Sciences, Peking University 12465, Beijing, China; 3 School of Life Science and Biopharmaceutics, Shenyang Pharmaceutical University 58575, Shenyang, China; 4 School of Basic Medicine and Clinical Pharmacy, China Pharmaceutical University 56651, Nanjing, China; University Children's Hospital Münster, Münster, Germany

**Keywords:** voriconazole, children, cut off value, trough concentration, invasive fungal disease, therapeutic drug monitoring, age groups, pharmacokinetics

## Abstract

There is limited evidence on the recommended voriconazole (VCZ) concentration ranges for prophylactic versus therapeutic use, and the factors influencing individualized dosing in young children remain insufficiently characterized. This retrospective study analyzed the clinical data from pediatric patients (2–17 years) at Peking University People’s Hospital from September 2020 to December 2024. The aim was to define optimal concentration thresholds for prophylaxis and treatment and identify sources of pharmacokinetic variability across age groups to support individualized dosing in children. In the exposure-effect study, VCZ trough concentration (C_min_) was significantly correlated with clinical efficacy. VCZ C_min_ ≥0.41 mg/L and C_min_ ≥1.115 mg/L were proven to be significant predictors of prophylactic and therapeutic dosing success, respectively. For pharmacokinetic analysis, patients were stratified into three age groups: Group 1 (2 to <6 years), Group 2 (6 to <12 years), and Group 3 (≥12 years). The final multiple regression analysis showed that affecting factors, including sex, moderate-to-severe inflammation, cytochrome P450 2C19 (CYP2C19) metabolic phenotype, and coadministration, explained 34.5%, 23.4%, and 47.6% of the variability in VCZ exposure in three groups, respectively. This study investigated pediatric patients with hematologic malignancies to define exposure-response differences between therapeutic and prophylactic VCZ regimens and identify age-related determinants of exposure variability. Overall, the findings support the use of individualized, age-appropriate dosing strategies to optimize VCZ exposure in children.

## INTRODUCTION

Invasive fungal disease (IFD) poses a serious clinical threat to immunocompromised pediatric patients, particularly those undergoing intensive chemotherapy, myelosuppressive therapy, or organ/hematopoietic stem cell transplantation (1). Voriconazole (VCZ) is recommended as the first-line therapy for the treatment or prophylaxis of IFD in multiple countries and is approved for use in children ≥2 years of age (2, 3). Due to its complex pharmacokinetics and significant interindividual variability, the efficacy and toxicity of VCZ are closely related to plasma drug concentrations (4, 5). Therefore, early implementation of precision antifungal therapy is crucial for improving outcomes.

To date, VCZ therapeutic drug monitoring (TDM) has been widely applied in clinical practice, the target trough concentration (C_min_) of which is usually fluctuated at 1.0–5.5 mg/L (lower limit 0.5–2 mg/L; upper limit 4–6 mg/L) (2, 6–9). However, current guidelines and expert consensus do not distinguish the concentration thresholds for different purposes of administration (therapeutic/prophylactic). Previous studies have reported that prophylactic therapy is effective in preventing the development of IFD, even when the patients’ blood levels remain consistently below the lower threshold (10). This phenomenon is similar to the concentration-effect relationship of posaconazole (prophylactic C_min_ ≥0.5 mg/L, therapeutic C_min_ >1 mg/L) (11), suggesting that VCZ may only be required at lower plasma concentrations to prevent IFD. Based on the available evidence, we hypothesize that the minimum effective concentration of VCZ to prevent IFD may be significantly lower than that required for treatment.

Systematic investigations of VCZ in specific populations, particularly pediatric patients, remain limited. Compared with adults, children exhibit distinct pharmacokinetic characteristics, with VCZ showing clear age-dependent behavior, including linear elimination in those younger than 12 years and substantially lower oral bioavailability, of approximately 46% (12–15). Additionally, physiological differences across various developmental stages in children further influence the pharmacokinetic characteristics of VCZ. Due to the immature gastrointestinal tract in newborns and infants, drug absorption occurs significantly more slowly compared with older children (13). The intestinal first-pass effect of the key metabolic enzyme cytochrome P450 3A4 (CYP3A4) exhibits marked age dependence. Its expression level peaks during the first year after birth. Subsequently, expression gradually declines with increasing age and stabilizes at lower levels during the school-age period (over 6 years old), directly influencing the systemic exposure of substrate drugs (16, 17). During distribution and metabolism, younger children exhibit lower plasma protein binding rates, potentially leading to elevated free drug concentrations (18). Infants and preschoolers have increased hepatic blood flow compared with older children; hence, hepatic clearance of the drug will be higher (19). Furthermore, CYP2C19 enzyme activity exhibits significant age dependence, reaching approximately 1.6 times the adult levels between ages 1 and 5 years. Subsequently, it gradually declines with age, approaching adult levels around the age of 10 years. This characteristic may be a key determinant of VCZ clearance (20).

To optimize individualized VCZ use in children, the pediatric population has been stratified by age to clarify dosing differences. Walsh et al. showed significant individual differences in VCZ plasma concentration-time profiles between the 2- to 5- and 6- to 11-year-old groups, consistent with clinical observations (14). Based on these physiologic characteristics, an age-stratified strategy for VCZ TDM is recommended: although existing studies (21–24) have elucidated VCZ pharmacokinetic variables in children aged 2–12 years, the underlying mechanisms explaining the differences in C_min_ between preschoolers and older children require further investigation. The developmental trajectories of metabolic enzymes, together with age-related changes in physiological parameters, suggest that the factors influencing VCZ C_min_ are age-specific. This variability is particularly pronounced in preschool children (2–5 years), highlighting the need for careful dose optimization and TDM in this age group.

This study investigated pediatric patients with hematologic malignancies in China, focusing on the following two objectives: first, to explore the relationship between plasma concentrations and therapeutic efficacy of VCZ during both therapeutic and prophylactic dosing, and to clarify the concentration threshold differences between these two regimens; second, to analyze the sources of variability in VCZ exposure across different age groups, considering both growth characteristics and pharmacokinetic differences. This study aims to provide scientific evidence for personalized, precision dosing of VCZ in pediatric patients and offer guidance to clinicians regarding dose adjustments and TDM.

## MATERIALS AND METHODS

### Patients

In this retrospective cohort study, we included pediatric patients aged 2–17 years who received VCZ at Peking University People’s Hospital from September 2020 to December 2024. Inclusion criteria included the following: meeting the diagnostic criteria for invasive fungal disease (refer to the European Organization for Research and Treatment of Cancer/Invasive Fungal Infections Cooperative Group and the National Institute of Allergy and Infectious Diseases Mycoses Study Group [EORTC/MSG] (25) and the Diagnostic Criteria and Principles of Treatment of Invasive Mycoses in Patients with Hematologic Malignancies, Sixth Revision) and receiving VCZ therapy; all children received VCZ according to standardized dosing regimens either intravenously or orally, and at least one steady-state C_min_ measurement was completed (after at least five consecutive doses).

Exclusion criteria included the following: (i) VCZ plasma concentration below the lower limit of quantification (LLOQ, 0.1 mg/L), (ii) combined use of potent inhibitors or inducers of CYP450 enzymes (e.g., rifampicin, phenytoin, and long-acting barbiturates), and (iii) missing important clinical data (including incomplete records of medication administration and ambiguous time of blood collection). The study protocol was approved by the Ethics Committee of Peking University People’s Hospital (Approval No. 2019PHB064-01), and all data collection complied with the ethical norms for clinical research. Written informed consent was obtained from all patients or legal representatives enrolled in the study.

Data on demographics, clinical diagnosis, VCZ dosage, VCZ C_min_, liver and renal function tests, laboratory values (C-reactive protein [CRP], total protein [TP], albumin [Alb], serum creatinine [SCr], gamma-glutamyl transpeptidase [γ-GT], and others), and concurrent medications (glucocorticoids [GCs], proton pump inhibitors [PPIs], and carbapenem) were all gathered on the same day as the monitoring of VCZ steady-state C_min_. The body surface area (BSA) of the children in this study was calculated by applying Stevenson’s formula (26). To counteract the impact of varying dosages on the steady-state C_min_ of VCZ, we used the C_min_ to body weight dose ratio (CDR)—defined as the ratio of C_min_ to daily dose per kilogram of body weight—to express the dose-corrected VCZ concentration (10^-1^·mg/L per mg/kg per day). Additionally, blood samples were collected within 1 h before the next administration for each patient. All laboratory measurements were performed on the same day and using the same platform at our hospital to ensure methodological consistency.

### Measurement of plasma VCZ C_min_

Applying a 1200 HPLC (Agilent, Germany) and liquid chromatography-tandem mass spectrometry (LC-MS/MS), the plasma concentrations of VCZ were determined. Chromatographic separation was achieved on a Hypersil Gold C18 column (2.1 × 50 mm, 1.9 μm) at 50°C with a flow rate of 0.5 mL/min, using acetonitrile containing 0.1% formic acid and water containing 0.1% formic acid as the mobile phase. Detection was performed on a QTRAP 5500 mass spectrometer (AB SCIEX) with electrospray ionization in multiple reaction monitoring mode. The assay showed good linearity over a concentration range of 0.01–10.00 mg/L (R² > 0.99), with intra- and inter-day precision <10% and accuracy within ±15%.

### Genotyping

Using a commercially available kit (E.Z.N.A.TM SQ Blood DNA Kit), patient genomic DNA was extracted from peripheral blood samples in accordance with standard protocols. For patients who underwent allogeneic hematopoietic stem cell transplantation, genotyping was conducted on peripheral blood samples collected before transplantation. Seven single-nucleotide polymorphisms (SNPs) in the *CYP2C19*, *CYP3A5*, *CYP3A4,* and *ABCB1* genes were selected based on dbSNP, HapMap, and PharmGKB databases, each with a reported minor allele frequency >5% in the Asian population. Genotyping was performed using the SNaPshot assay, using standard PCR and capillary electrophoresis procedures. CYP2C19 phenotypes were classified as poor metabolizers (PM), intermediate metabolizers (IM), or normal metabolizers (NM) according to the Clinical Pharmacogenetics Implementation Consortium standards (27).

### Outcome assessment

The definition and treatment response of IFD were in accordance with the EORTC/MSG (1, 25, 28). For targeted antifungal therapy, a complete or partial response at the end of treatment constituted treatment success, whereas a stable response, disease progression, or IFD-related death was classified as treatment failure. Successful diagnosis-driven or empirical therapy required fulfillment of all criteria from initiation until 7 days after discontinuation, including survival without breakthrough infection, no treatment discontinuation due to adverse events or inefficacy, and resolution of fever during neutropenia. Success for antifungal prophylaxis required no breakthrough infection during and up to 7 days post-therapy without VCZ discontinuation due to adverse events.

### Statistics

Statistical analyses were carried out using SPSS software version 27.0. Categorical variables were summarized as frequencies and percentages, whereas continuous variables with non-normal distributions were reported as medians and interquartile ranges (IQR). For univariate analysis, correlations of non-normally distributed continuous variables were assessed using Spearman’s rank correlation test. The Mann-Whitney U test and Kruskal-Wallis tests were used to compare differences in constant values between different groups. Categorical variables were analyzed using χ^2^ tests or Fisher’s exact tests. Graphs were output using GraphPad Prism software (version 9.5.0).

#### Exposure-prognosis correlation and threshold determination analysis

Pharmacodynamic analysis uses “treatment events” as independent units of study. Given that most pediatric patients with hematologic malignancies have compromised immune function, fungal infections may recur frequently; therefore, each distinct treatment event is included separately in the analysis. For each event, the median of all available C_min_ values was calculated, and the corresponding efficacy endpoints were included in the statistical analysis. Patients were divided into two groups according to the purpose of medication: preventive and therapeutic medication groups. Efficacy outcomes (success or failure) were summarized using descriptive statistics and reported as frequencies and percentages (n, %) for each study group. The relationship between VCZ C_min_ and efficacy in both groups was statistically and analytically analyzed. Thresholds for VCZ C_min_ (therapeutic levels) were derived from the analysis of subjects’ work characteristic curves.

#### Descriptive pharmacokinetic parameter analysis

Each SNP was tested for Hardy-Weinberg equilibrium (HWE) compliance using χ^2^ tests, with a *P*-value < 0.05 considered evidence of significant deviation. In order to fit into clinical practice, CRP was included as a categorical variable to compare the impact of CRP on VCZ C_min_ between no/mild inflammation (<40 mg/L) and moderate inflammation (40–200 mg/L).

Patients were divided into three groups (2 to <6; 6 to <12; and ≥12 years old). Within each group, univariate analyses were first performed to identify candidate factors associated with CDR, followed by multivariate linear regression including variables with *P* < 0.1. Differences among the three groups were analyzed using the Kruskal-Wallis test, followed by post hoc pairwise comparisons with Bonferroni correction. To satisfy the assumption of homogeneity of variance for multiple linear regression, the dependent variable CDR was log-transformed (lgCDR). To reduce small-sample bias, only combination therapies comprising more than 10% of the total cases were compared between groups.

## RESULTS

### Exposure-response analysis

#### Baseline characteristics of patients

**Fig 1:**
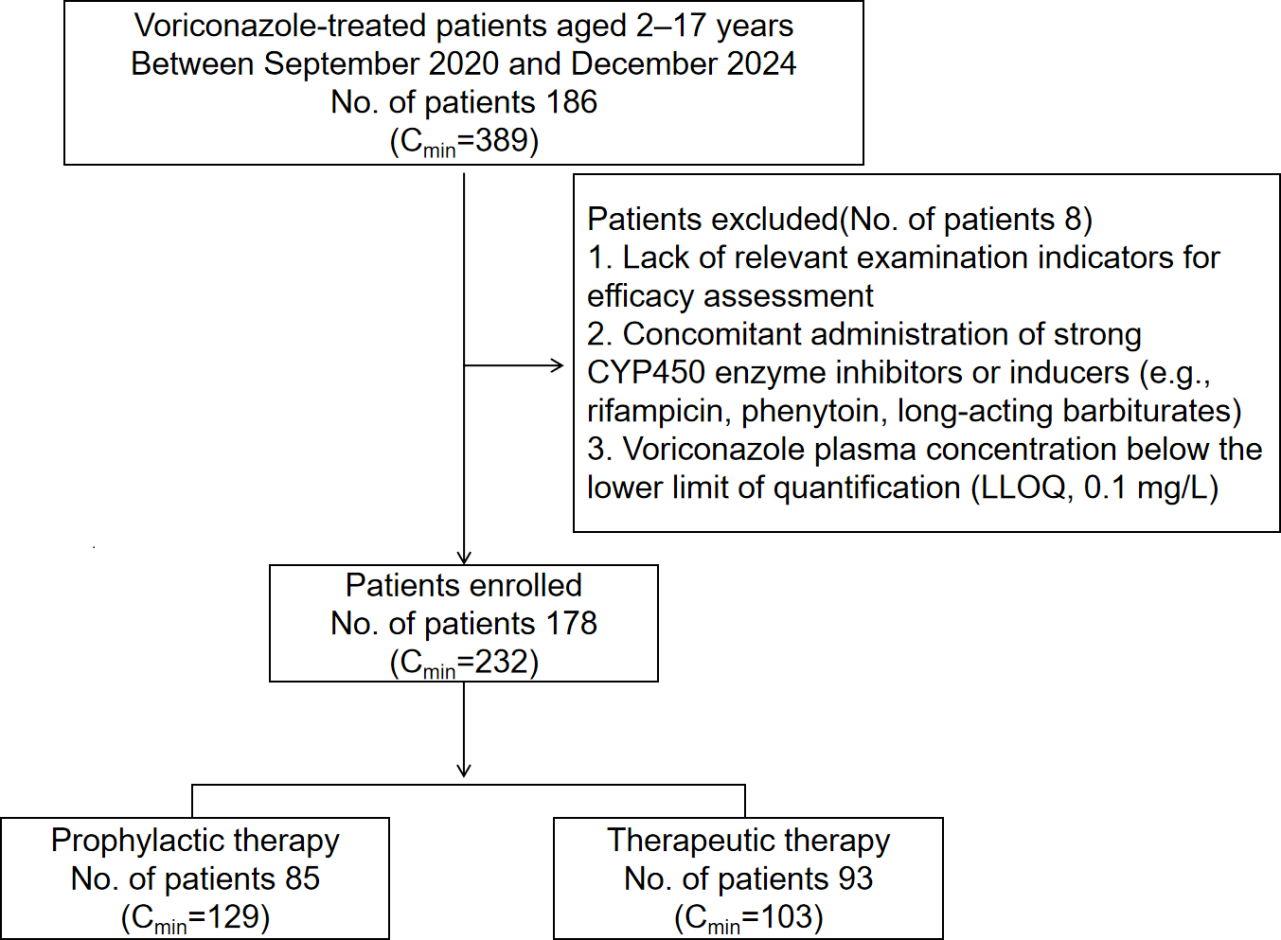
Flowchart of patient selection for efficacy analysis.

After excluding cases in which the efficacy could not be assessed due to the lack of relevant examination indices or clinical data for efficacy assessment, 178 children were finally included in this part of the study (Fig. 1). Patients were divided into prophylactic (*n* = 85) and therapeutic (*n* = 93) groups based on treatment purpose. Acute myeloid leukemia was the most common underlying disease in both groups. No significant differences in baseline characteristics were observed between the two groups. Detailed baseline characteristics are presented in Table 1.

**TABLE 1:** The characteristics of basic information in the two groups^*a*^

Parameters	No. of patients (%) or median [IQR]	
	Prophylactic therapy	Therapeutic therapy
No. of patients	85	93
Age (yr)	8.0 [6.0, 12.0]	9.0 [5.0, 14.0]
Sex, Male (no. %)	48 (56.5)	57 (61.3)
BMI (kg/m^2^)	15.8 [14.5, 17.4]	15.8 [14.4, 18.9]
WT (kg)	26.0 [19.0, 43.5]	25.6 [18.0, 49.9]
Patient’s underlying condition (no. %)		
AML	49 (57.6)	44 (47.3)
ALL	22 (25.9)	33 (35.4)
Other hematological malignancy	7 (8.2)	8 (8.6)
Other	7 (8.2)	8 (8.6)

^*a*^ BMI, body mass index; WT, weight; NEUT, neutrophil; AML, acute myeloid leukemia; and ALL, acute lymphocytic leukemia.

#### Trough concentration and administered dose

**Fig 2:**
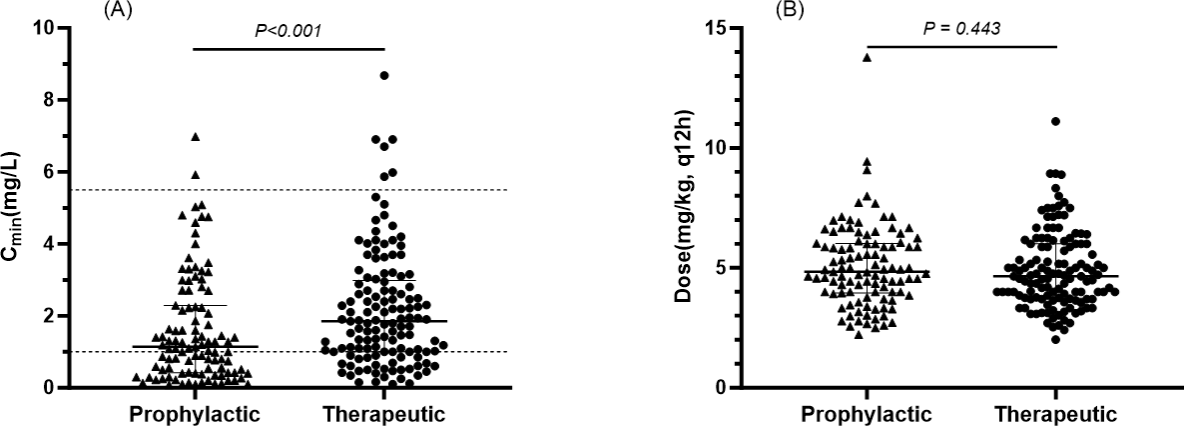
(**A**) Scatter dot plot of the initial C_min_ in the two groups. The region between horizontal reference lines represents the therapeutic range (1.0–5.5 mg/L). (**B**) Scatter dot plot of the single weight-normalized dosage in the two groups.

A total of 232 VCZ trough samples were analyzed. Prophylactic VCZ C_min_ was significantly lower than that in the therapeutic group (*P < 0.001*). Dose distributions were comparable between the two groups. Detailed results are shown in Fig. 2.

#### Trough concentration and efficacy

**Fig 3:**
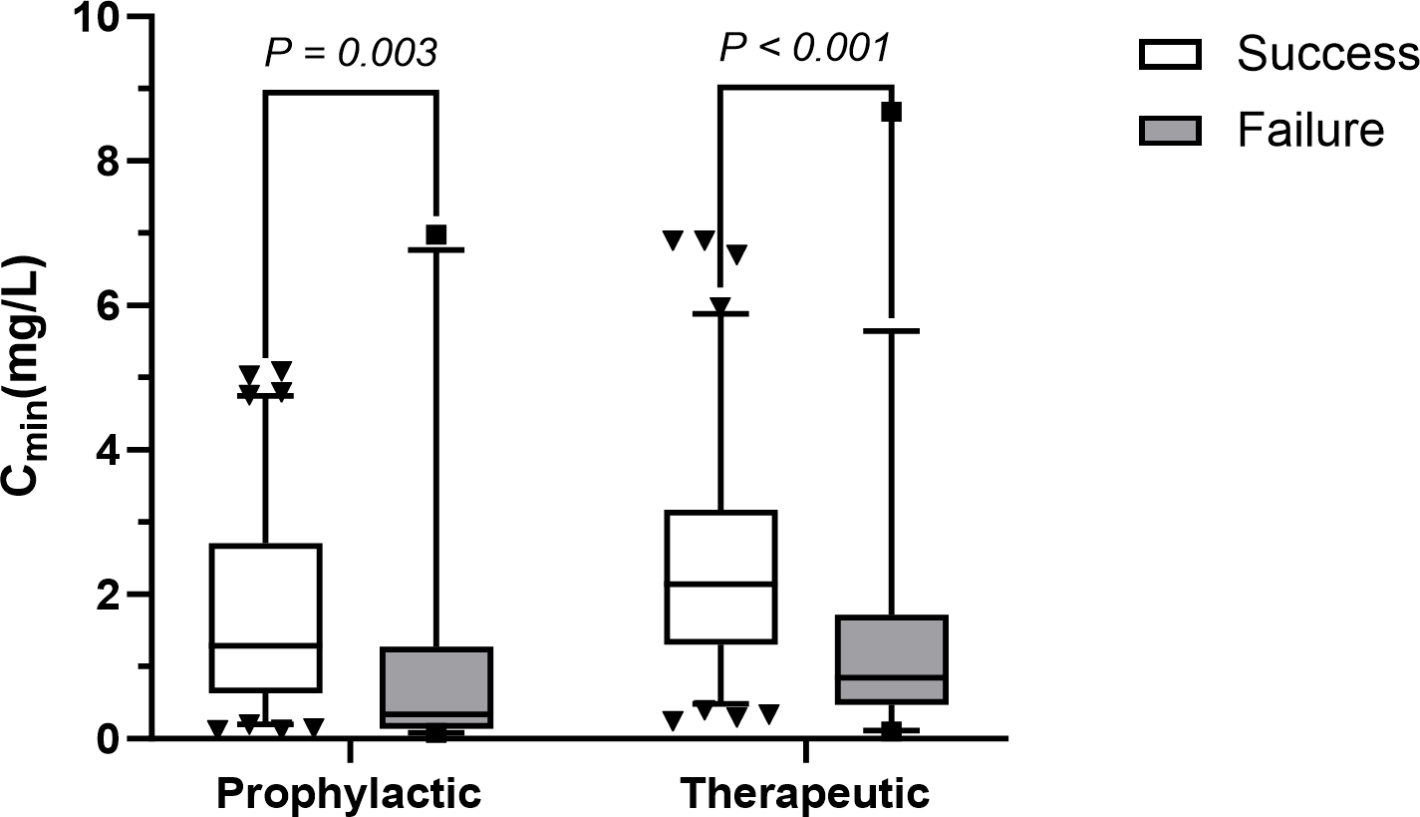
Distribution of C_min_ under VCZ treatment success and failure in the two groups.

Preliminary analysis of baseline characteristics revealed that the VCZ C_min_ was significantly different between the success and failure groups in both the prophylaxis (Table S1) and treatment (Table S2) cohorts. Overall efficacy was 75.9%, with no significant difference between the prophylactic and therapeutic groups (*P > 0.05*). Higher VCZ C_min_ values were significantly associated with successful outcomes in both groups (*P = 0.003* and *P < 0.001*, respectively). See Fig. 3 for details. The only other statistically significant variable was patient age in the treatment group, with a mere 2-year difference deemed clinically irrelevant (Table S2).

**Fig 4:**
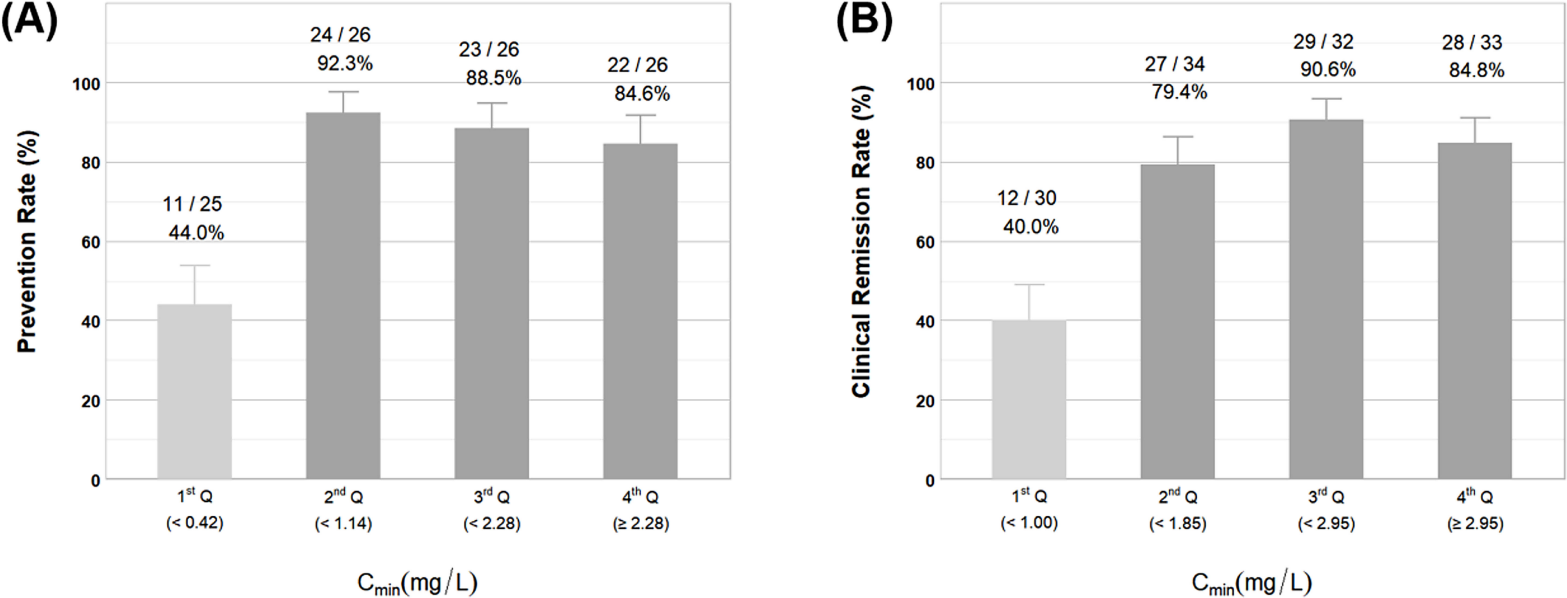
(**A**) Graph of prevention success rates in quartiles of the prevention group. (**B**) Graph of clinical remission rates in quartiles of the treatment group.

Figure 4 displays the findings of the interquartile analysis of the relationship between VCZ C_min_ and efficacy. According to the interquartile analysis, a strong dose-response relationship was identified between serum C_min_ and clinical outcomes. Based on VCZ C_min_ quartile grouping, efficacy in both the prophylaxis and treatment groups significantly improved from the first quartile to the second quartile, subsequently stabilizing at higher levels. Efficacy differed significantly across concentration categories in both the prophylactic (Pearson χ² = 22.022, *P* < 0.001; χ² for trend = 9.838, *P* = 0.002) and therapeutic groups (Pearson χ² = 25.414, *P* < 0.001; χ² for trend = 16.529, *P* < 0.001), demonstrating a clear concentration-response relationship.

**Fig 5:**
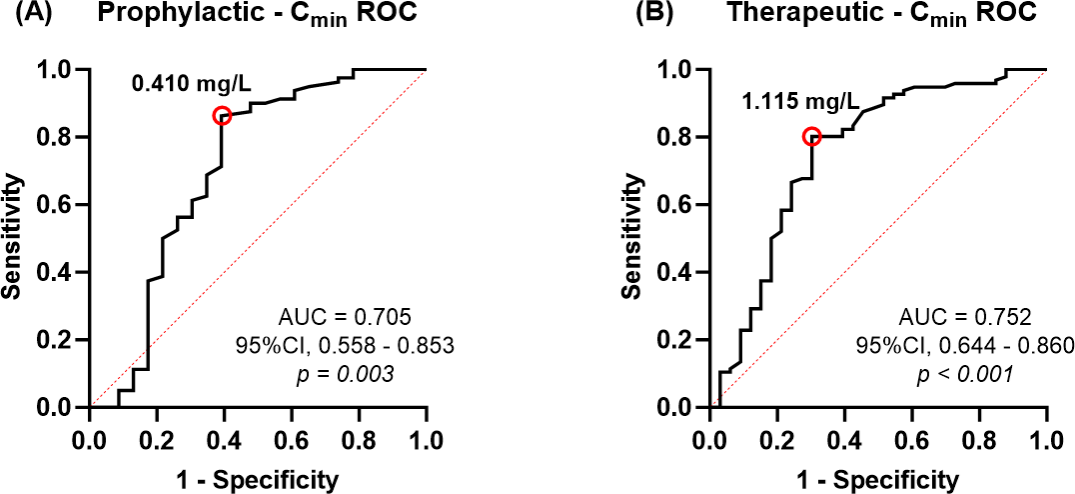
(**A**) Receiver operating characteristic curve for C_min_ levels with regard to the occurrence of prophylactic success. (**B**) Receiver operating characteristic curve for C_min_ levels with regard to the occurrence of treatment success.

ROC curve analysis demonstrated a significant correlation between VCZ C_min_ and clinical efficacy. For prophylactic use, the analysis revealed an area under the curve (AUC) of 0.705, with Youden index analysis identifying 0.410 mg/L as the optimal C_min_ threshold. In therapeutic applications, the ROC analysis showed an AUC of 0.752, with 1.115 mg/L established as the optimal C_min_ cutoff. The detailed results of the ROC analysis are illustrated in Fig. 5.

### Pharmacokinetic analysis

#### Baseline characteristics of patients

**Fig 6:**
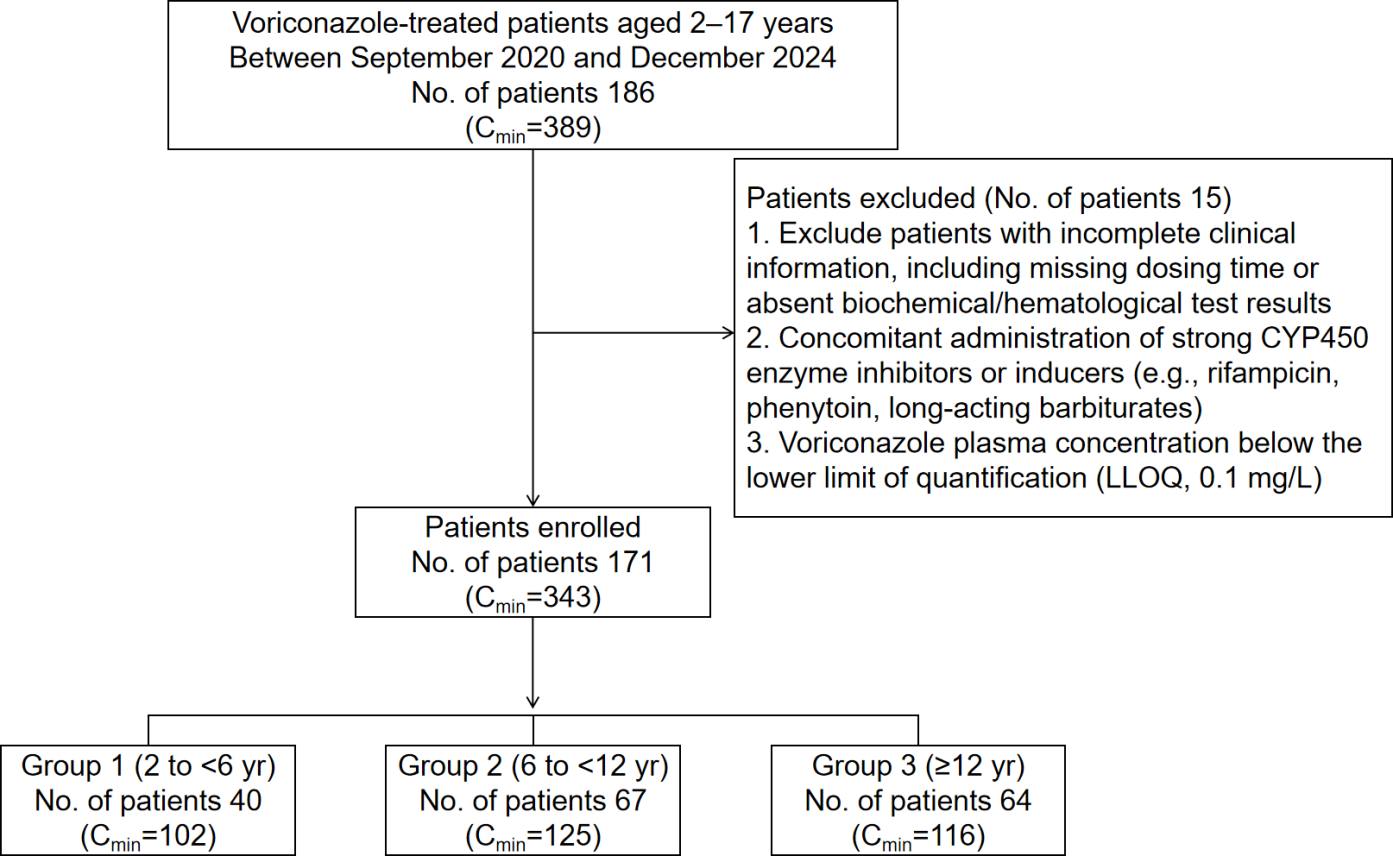
Flowchart of patient selection for pharmacokinetic analysis.

A total of 171 pediatric patients were ultimately eligible for the trial. These patients (103 females and 68 males) were divided into three groups based on their age (Group 1: 2 to <6 years, Group 2: 6 to <12 years, and Group 3: ≥12 years) (Fig. 6). Table 2 shows the patients’ characteristics.

**TABLE 2:** Demographic and clinical characteristics of patients

	No. of patients (%) or median [IQR]				
Parameters	Total (*n* = 171）	Group 1: 2 to <6 yr (*n* = 40）	Group 2: 6 to <12 yr (*n* = 67）	Group 3: ≥12 yr (*n* = 64）	*P* Value
Demographics					
Age (yr)	10.0 [6.0, 14.0]	3.0 (3, 5)	8.0 [8, 10.5]	14.0 (14, 16)	<0.001
Sex, Male (no. %)	103 (60.2)	21 (52.5)	35 (52.2)	47 (73.4)	0.024
WT (kg)	32.0 [20.0, 49.0]	15.0 [14.5, 20.5]	25.0 [17.3, 41.3]	53.0 [48.5, 67.0]	<0.001
BMI (kg/m^2^)	16.0 [14.6, 18.4]	15.2 [14.4, 16.8]	15.0 [13.8, 17.2]	18.3 [17.1, 22.3]	<0.001
HSCT (no. %)	27 (15.8)	7 (17.5)	11 (16.4)	9 (14.0)	
Patient’s underlying condition (no. %)					0.085
AML	89 (52.0)	23 (57.5)	32 (47.8)	34 (53.1)	
ALL	57 (33.3)	14 (35.0)	21 (31.3)	22 (34.4)	
Other hematological malignancy	13 (7.6)	1 (2.5)	9 (13.4)	3 (4.7)	
Other	12 (7.0)	2 (5.0)	5 (7.5)	5 (7.8)	
Treatment indication (no. %)					0.251
Therapeutic	44 (25.7)	9 (22.5)	18 (26.9)	17 (26.6)	
Empirical	32 (18.7)	5 (12.5)	16 (23.9)	11 (17.2)	
Prophylactic	95 (55.6)	26 (65.0)	33 (49.2)	36 (56.2)	
CYP2C19 metabolizer phenotype (no. %)					0.611
NM	68 (39.8)	14 (35.0)	28 (41.8)	26 (40.6)	
IM	74 (43.3)	17 (42.5)	27 (40.3)	30 (46.9)	
PM	28 (16.4)	9 (22.5)	12 (17.9)	7 (10.9)	
UM	1 (0.6)	–	–	1 (1.6)	
Administration route during sampling (no. %)					0.005
Intravenous	196 (56.2)	71 (69.6)	67 (53.6)	56 (48.3)	
Oral	153 (43.8)	31 (30.4)	58 (46.4)	60 (51.7)	
VCZ dose (mg/kg)	4.7 [3.9, 6.1]	6.3 [3.2, 9.1]	5.0 [2.5, 8.3]	3.7 [1.9, 6.5]	<0.001
Concomitant medications (no. %)					
PPI	65 (18.6)	16 (15.7)	20 (16.0)	29 (25.0)	0.124
GC	215 (61.6)	58 (56.9)	74 (59.2)	80 (69.0)	0.140
Carbapenems	139 (39.8)	34 (33.3)	46 (36.8)	58 (50.0)	0.027
CsA	98 (28.1)	25 (24.5)	42 (33.6)	30 (25.9)	0.247
SMZ/TMP	94 (26.9)	24 (23.5)	39 (31.2)	29 (25.0)	0.372
Acyclovir	74 (21.2)	18 (17.6)	32 (25.6)	23 (19.8)	0.310
MMF	34 (9.7)	13 (12.7)	12 (9.6)	9 (7.8)	0.465
Laboratory parameter^*a*^					
CRP (mg/L)	5.1 [1.0, 22.6]	5.1 [0.9, 22.4]	4.2 [1.1, 16.2]	13.2 [1, 43.6]	0.010
<40 mg/L	274 (82.6)	81 (82.7)	107 (87.8)	86 (77.1)	0.105
≥40 mg/L	72 (17.4)	21 (17.3)	18 (12.2)	33 (22.9)	
Alb (g/L)	37.0 [33.7, 40.4]	37.2 [34.2, 41.2]	36.5 [33.3, 39.1]	35.1 [31.6, 39.2]	0.028
TP (g/L)	64.5 [58.3, 68.9]	64.9 [60, 68.7]	60.9 [54.9, 67.9]	58.9 [53.4, 65.8]	<0.001
ALT (U/L)	20.0 [12.0, 36.0]	16 (10, 37)	22 [13.5, 34.5]	26 (13, 43)	0.074
AST (U/L)	22.0 [15.0, 32.0]	23 (17, 36)	20 [12.5, 25]	17 [12, 28.5]	<0.001
γ-GT (U/L)	38.0 [23.0, 68.0]	22 [17, 48]	30 [20.5, 56]	50 [30.5, 112.5]	<0.001
ALP (U/L)	110 [78.0, 161.0]	131 [84, 159]	96 [74.5, 133]	81 [65.5, 136]	<0.001
T-BIL (μmol/L)	8.4 [5.9, 11.8]	7.9 [5, 10.2]	8.1 [6, 12.2]	10 [8.1, 13.8]	<0.001
SCr (μmol/L)	32.0 [25.0, 42.0]	27 (22, 33)	34 (30, 40)	49 [40, 58.5]	<0.001
GFR (mL/(min·1.73m²))	179.2 [158.7, 201.7]	199.3 [178.3, 225.2]	173.3 [160.2, 190.6]	150.6 [140.5, 163.6]	<0.001

^*a*^ PPI, proton pump inhibitor; GC, glucocorticoid; CsA, cyclosporine A; SMZ/TMP, compound sulfamethoxazole; MMF, mycophenolate mofetil; CRP, C-reactive protein; Alb, albumin; TP, total protein; ALT, alanine transaminase; AST, aspartate aminotransferase; γ-GT, gamma-glutamyl transpeptidase; ALP, alkaline phosphatase; T-BIL, total bilirubin; SCr, serum creatinine; and GFR, glomerular filtration rate. –, no patients were present in this category, rather than missing data.

#### Distribution of plasma VCZ C_min_ and maintenance dosage

**Fig 7:**
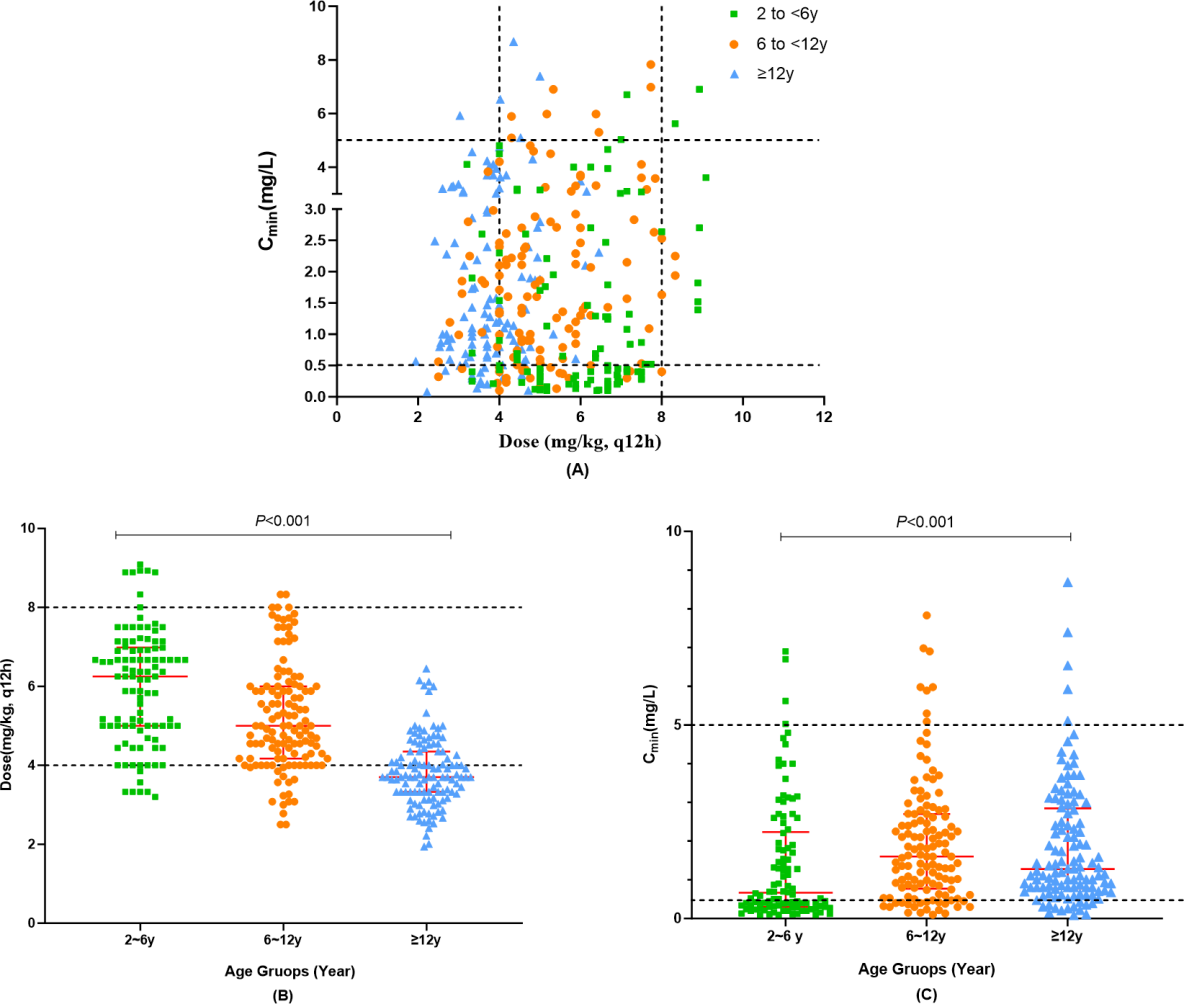
(**A**) Overall VCZ C_min_ scatter plot at various weight-normalized dosages. The therapeutic range (0.5–5.0 mg/mL) is shown by the space between the horizontal lines. (**B**) Scatter dot plot of the single weight-normalized dosage among three age groups. (**C**) Scatter dot plot of initial C_min_ in the three age groups. The median and interquartile range are shown by each error bar.

The study analyzed 343 samples, with a median C_min_ of 1.28 mg/L (range: 0.08–8.68), of which 71.3% (*n* = 249) were within the therapeutic range (0.5–5.0mg/L) and 23% (*n* = 79) were subtherapeutic. Figure 7A depicts the distribution of measured VCZ C_min_ and associated dosages.

Significant dose variations were observed among groups (Fig. 7B). Weight-adjusted dosing showed no correlation with VCZ trough levels in Groups 1 and 3. However, a predictable relationship between initial weight-adjusted dose and C_min_ was observed in patients of 6 to <12 years old (*n* = 125, coefficient = 0.236, *P = 0.008*).

The VCZ C_min_ distribution between the three age groups of children is shown in Fig. 7C and Table 3. The median (range) VCZ C_min_ differed significantly among the three groups (*P < 0.001*). The frequency of VCZ C_min_ within the therapeutic range was significantly lower in Group 1 than in Groups 2 and 3 (c_1_^2^ = 12.485, *P<0.001*; c_2_^2^ = 17.461, *P < 0.001*), with no significant difference between Groups 2 and 3. Similarly, the proportion of children with subtherapeutic concentrations declined with age, decreasing from 43.1% in the 2 to <6-year group to 16.8% in the 6 to <12-year group *(P < 0.001)*.

**TABLE 3:** VCZ C_min_ differences and concentration attainment among three age groups

	No. of samples (%) or median [interquartile range, IQR]			
Variables	2 to <6 years	6 to <12 years	≥12 years	*P* Value
C_min_ (mg/L)	0.67 [0.30, 2.23]	1.60 [0.77, 2.70]	1.28 [0.77, 2.85]	<0.001
<0.5 (n, %)	44 (43.1)	21 (16.8)	14 (12.1)	
0.5–5.0 (n, %)	54 (52.9)	96 (76.8)	97 (83.6)	
>5.0 (n, %)	4 (3.9)	8 (6.4)	4 (3.4)	

#### Variability of VCZ exposure among the three groups

**Fig 8:**
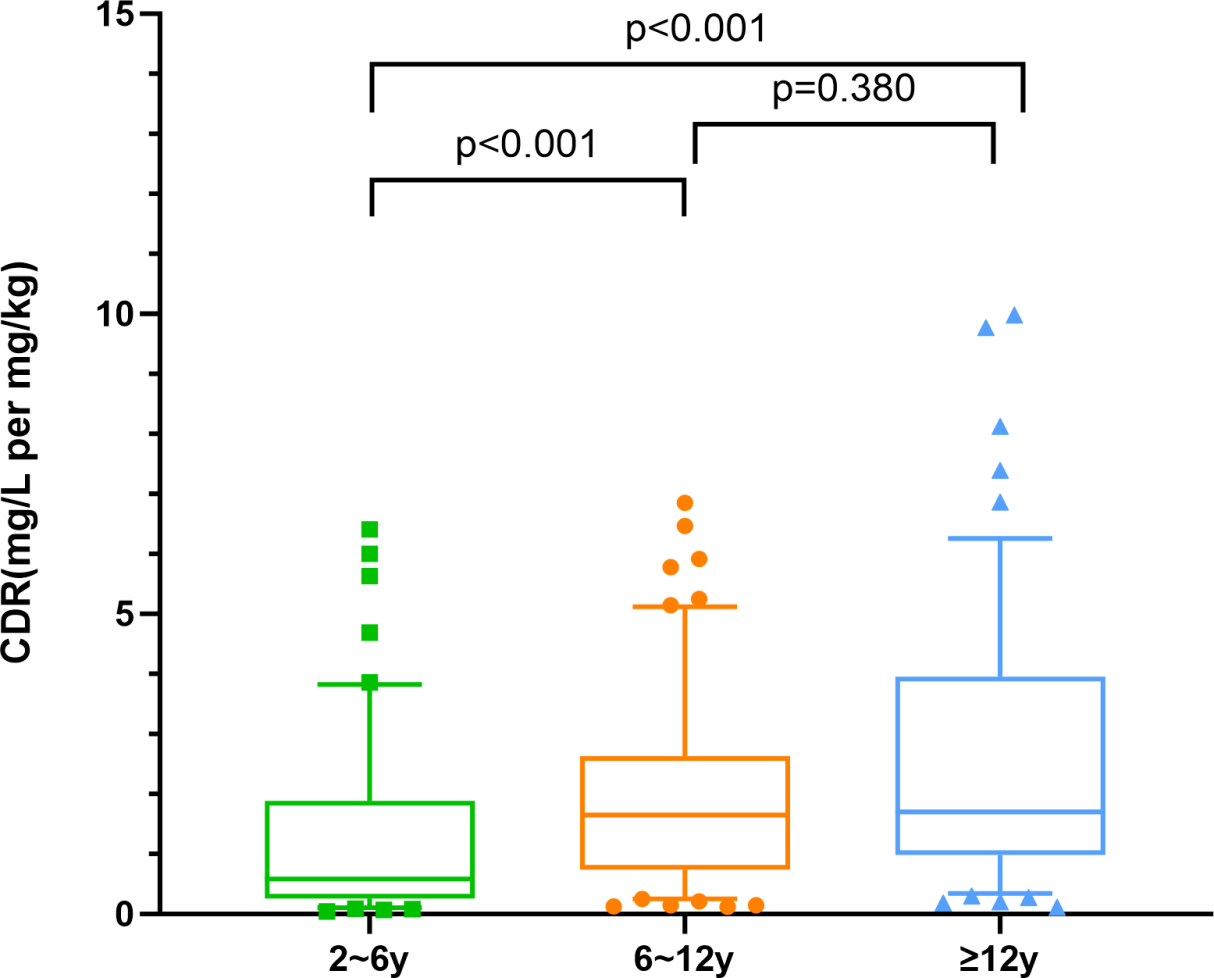
Comparison of the CDR of VCZ concerning the differences among the three age groups. The boxplot displays the median (central line) and interquartile range (25th to 75th percentiles), while the whiskers extend to the 5th and 95th percentiles. *P* values were adjusted for multiple comparisons using the Bonferroni correction.

The VCZ CDR showed significant interindividual variability (0.04–9.98 mg/mL/mg/kg). Figure 8 shows that Group 1 (2 to <6 years) had considerably lower CDR compared to Groups 2 (6 to <12 years) and 3 (≥ 12 years) (*P <* 0.001), while there was no significant difference in CDR between Groups 2 and 3 *(P* = 0.380).

**Fig 9:**
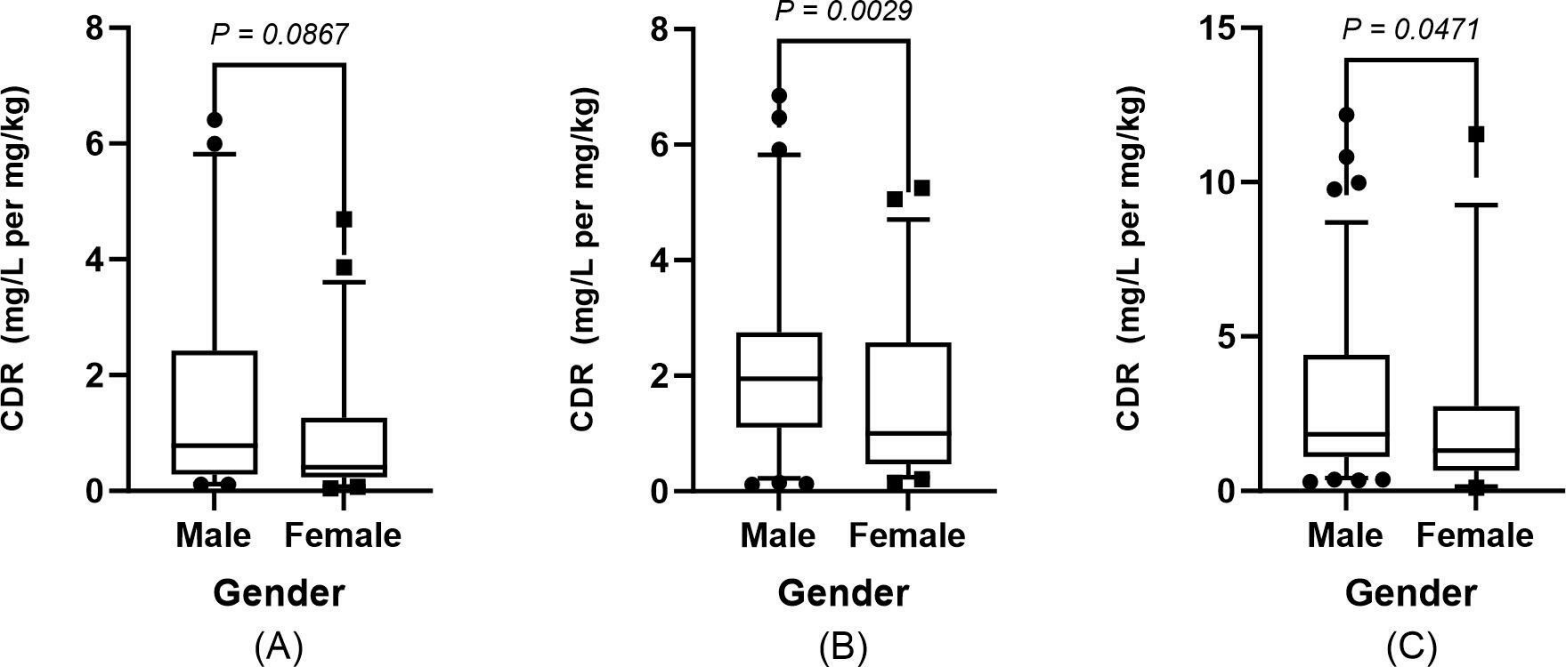
Sex influence on CDR of VCZ among three age groups. (**A**) Males had higher CDR than females, but the difference was not significant in Group 1 (2 to <6 years). (**B**) Males exhibited a considerably greater CDR than females in Group 2 (6 to <12 years). (**C**) Males exhibited considerably higher CDR than females in Group 3 (≥ 12 years). Boxplots represent median values (center line), interquartile ranges (boxes), and 5th–95th percentiles (whiskers).

Univariate analyses were performed to assess the effects of demographic, pathophysiological, and genetic factors on steady-state VCZ C_min_ across age subgroups (Tables S3 and S4). The results showed that in the 2–6 years and ≥12 years age groups, patients with CRP ≥40 mg/L had significantly higher CDR than those with CRP <40 mg/L (*P* < 0.05); In the ≥6-year-old group, males had higher CDR than females, with a more pronounced difference in the 6 to <12-year-old subgroup (*P* = 0.002) (Fig. 9). In the 2 to <12-year-old group, patients receiving concomitant carbapenem therapy had higher CDR than those who did not receive concomitant carbapenem therapy (*P* < 0.05), and those receiving concomitant PPI therapy in the 2 to <6-year-old subgroup had higher CDR than those who did not receive concomitant PPI therapy (*P* = 0.018). In the ≥12-year-old group, CDR was lower in those receiving concomitant glucocorticoids than in those not receiving them (*P* = 0.002). Genotype analysis showed that in the 2 to <12-year-old group, CYP2C19*PM had higher CDR than NM (*P* < 0.001). In the 2 to <6 years group, ABCB1 rs1045642 GG genotype had lower CDR than AA and GA (*P*<0.01). In the 6 to <12 years group, CYP3A4 rs4646437 CC genotype had higher CDR than TT (*P*=0.034, *P*=0.030). In the ≥12 years group, CYP3A4 rs2242480 TT genotype and CYP3A5 rs776747 TT genotype both had lower CDR than the corresponding wild-type and heterozygous genotypes (*P* < 0.01).

Multiple linear regression was performed to identify the different factors associated with VCZ C_min_ corrected for dose in three age groups (Table 4).

**TABLE 4:** Multiple linear regression analysis for VCZ lgCDR (mg/L per mg/kg)^*e*^

Parameters	Group 1 (2 to <6 yr) R^2^ = 0.345				Group 2 (6 to <12 yr) R^2^ = 0.234			Group 3 (≥12 yr) R^2^ = 0.476		
	Coefficient		SE	*P* value	Coefficient	SE	*P* value	Coefficient	SE	*P* value
Sex	−0.013		0.102	0.903	−0.162^*a*^	0.071	0.025	−0.217^*a*^	0.071	0.003
BSA	0.792		0.463	0.090	0.243	0.158	0.127	/^*f*^	/	/
CRP (mg/L) <40 (ref.) ≥40	0.214		0.117	0.070	0.021	0.103	0.839	0.318^*a*^	0.072	<0.001
PPIs use	0.106		0.130	0.416	/	/	/			
Carbapenems use	0.190^*a*^		0.095	0.048	0.159^*a*^	0.069	0.023	−0.023	0.064	0.721
Glucocorticoid use	/		/	/	/	/	/	−0.215^*a*^	0.070	0.003
CYP2C19 phenotype^*b*^	IM	0.017	0.105	0.871	0.184^*a*^	0.072	0.012	0.196^*a*^	0.065	0.003
	PM	0.418^*a*^	0.137	0.003	0.442^*a*^	0.105	<0.001	0.356^*a*^	0.102	0.001
ABCB1 (rs1045642) genotype^*c*^	GA	0.253^*a*^	0.099	0.012	/	/	/	/	/	/
	AA	0.314^*a*^	0.142	0.030	/	/	/	/	/	/
CYP3A5*3 (rs776746) genotype^*d*^	CT	/	/	/	/	/	/	−0.137^*a*^	0.063	0.031
	TT	/	/	/	/	/	/	−0.759^*a*^	0.128	<0.001
Alb	/		/	/	/	/	/	−0.013	0.007	0.052
Constant value	−1.067		0.346	0.003	−0.261	0.178	0.151	0.862	0.263	0.001

^*a*^ The variable is significant, at the level of 0.05 (double tail).

^*b*^ The phenotype of CYP2C19 was set as a dummy variable, and NM is the reference variable.

^*c*^ The ABCB1 (rs1045642) variant was set as dummy variable, and the GG genotype is the reference variable.

^*d*^ The CYP3A5*3 (rs776746) variant was set as dummy variable, and the CC genotype is the reference variable.

^*e*^ BSA, body surface area; CRP, C-reactive protein; IM, intermediate metabolizer; PM, poor metabolizer; and NM, normal metabolizer.

^*f*^ /, variable was not included in the multiple linear regression model.

##### Patients 2 to <6 years

This group included 40 patients, providing 102 samples. In multivariate regression analysis, variables such as sex, CRP, CYP2C19 phenotype, ABCB1 (rs1045642) genotype, and carbapenem comedication were included in the final model, as shown in Table 4. Of these, only CYP2C19 phenotype, ABCB1 (rs1045642) genotype, and carbapenem comedication did have a significant effect on VCZ CDR. The linear relationship between VCZ CDR and the independent variables included in the model was all significant (F = 14.299, *P* < 0.001). The model explained 34.5% of the variation in VCZ CDR.

##### Patients 6 to <12 years

This group consisted of 125 samples collected from 67 patients. In multivariate regression analysis, sex, CYP2C19 phenotype, and carbapenems comedication did show a significant effect and remained in the final model. The linear association between VCZ CDR and the independent variables included in the model was all significant (F = 12.794, *P* < 0.001). The model explained 23.4% of the variation in VCZ CDR.

##### Patients ≥12 years

This group consisted of 116 samples collected from 64 patients. Sex, CRP, CYP2C19 phenotype, CYP3A5*3 genotype, and GCs comedication showed a significant effect on VCZ CDR in multivariate regression analysis and remained in the final regression model as shown in Table 4. The linear relationship between VCZ CDR and these independent variables included in the model was all significant (F = 16.884, *P* < 0.001). The model explained 47.6% of the variation in VCZ CDR.

In summary, our study demonstrates that the pharmacokinetic characteristics of VCZ differ significantly across age groups in pediatric patients (Table 4). CYP2C19 phenotype was a key covariate for CDR across all ages and was included in the final model. Genetic effects were age-specific: ABCB1 (rs1045642) correlated with CDR in 2- to 6-year-old patients (*P* < 0.05), while CYP3A5*3 affected those ≥12 years (*P* < 0.05). Drug interactions were also age-dependent: carbapenems lowered CDR in children <12 years (*P* < 0.05), and glucocorticoids were associated with reduced CDR only in the ≥12-year group (*P* < 0.01). CRP levels significantly influenced CDR in the ≥12-year group (*P* < 0.001).

## DISCUSSION

This study is among the few pharmacokinetic and pharmacodynamic analyses of VCZ based on real-world data in Chinese children. It clearly distinguished effective thresholds under different therapeutic purposes and systematically analyzed key factors influencing VCZ concentrations across age groups, providing important insights for optimizing individualized dosing strategies in pediatrics.

There is no consensus on the target range of VCZ therapeutic concentration, and most studies suggest a lower limit of >0.5 mg/L or >1 mg/L to improve efficacy (2, 9), but thresholds generally do not differentiate between prophylactic and therapeutic use. In this study, we found that C_min_>0.41 mg/L is the optimal threshold for prevention of IFD, whereas the therapeutic threshold should be increased to >1.115 mg/L. Although our findings are largely consistent, certain discrepancies remain. Compared with the literature, the therapeutic threshold in this study was slightly higher, potentially due to pharmacokinetic characteristics specific to pediatric patients, such as faster metabolic clearance, different body surface area/body weight ratios, and higher and fluctuating enzyme activity. Furthermore, the study included severely immunosuppressed pediatric patients at high risk of fungal infections. The prophylactic dose was lower than that reported in the literature, potentially attributable to robust monitoring systems, environmental controls, and preventive management measures, which maintained a low breakthrough rate even at lower plasma concentrations. Considering children’s sensitivity to hepatic, cutaneous, and developmental effects, prophylactic therapy with >0.41 mg/L effectively balances efficacy and safety.

The C_min_ of VCZ in the prophylaxis group was significantly lower than in the treatment group. Although the single dose was slightly higher, this difference may be due to faster metabolism and a larger volume of distribution in younger children. The prophylaxis group included a greater proportion of younger children, leading to persistently low plasma concentrations despite higher weight-adjusted doses. VCZ exposure is also affected by multiple factors, including genotype, inflammation, and concomitant medications. To better understand the sources of exposure variability, we conducted additional pharmacokinetic studies to support individualized pediatric dosing.

VCZ exposure was significantly lower in younger children, with about half of those aged 2–6 years not reaching the target C_min_ compared to ~15% in older groups, indicating a higher risk of underexposure in younger children. This study indicates that children younger than 6 years of age may require higher doses than currently recommended, consistent with Kato et al. (29). The median dose required to achieve therapeutic concentrations in children younger than 5 years of age has been shown to be much higher than the current recommendations (30, 31). In this study, VCZ C_min_ and weight-corrected dose were positively correlated in children aged 6 to <12 years but not in the 2 to <6 years group, likely due to age-related differences in hepatic clearance and VCZ pharmacokinetics (14, 15). Given the complex pharmacokinetics of VCZ in the population and the difficulty in predicting C_min_ (14), dose adjustment in children 2–5 years of age needs to be more cautious.

Regression analysis showed higher VCZ exposure in male children aged ≥6 years than in females, consistent with adult findings reported by Allegra et al. (32). However, investigations in adults aged 18–45 years revealed higher C_min_ in females (33). Potential explanations for these gender differences include variations in CYP enzyme activity, the influence of sex hormones on drug absorption and distribution, and differences in body fat percentage between males and females. Given the moderate lipophilicity of VCZ, the higher body-fat proportion in women may partly account for their lower plasma concentrations. Furthermore, these physiological factors undergo significant changes during different developmental stages in children, suggesting that the pattern of gender-related effects on VCZ exposure may differ from that observed in adults. In summary, gender should be fully considered in high-risk pediatric patients, particularly the risk of under-exposure in females, to optimize dosing.

In this study, CRP ≥40 mg/L was used to assess the degree of inflammation (24). The results showed that moderate-to-severe inflammation significantly increased the dose-adjusted C_min_ of VCZ in children aged ≥12 years, whereas no such association was observed in children aged 2–11 years, consistent with previous findings (24, 30, 34). CRP may affect VCZ metabolism by downregulating CYP2C19 expression and activity (35). However, in preschoolers, higher hepatic blood flow (19) and CYP2C19 activity (20) may have attenuated the inhibitory effect of inflammation, thus explaining the lack of significant correlation in this age group.

Controversy remains regarding the clinical significance of CYP2C19 genotype on VCZ pharmacokinetics in pediatric patients (36). The present study further supports the notion that CYP2C19 polymorphisms are strongly associated with high variability in VCZ C_min_, with IMs and PMs generally exposed at higher levels than NMs, in line with previous studies (14, 15, 36). The higher VCZ clearance in younger children may be related to their relatively greater liver weight or differences in CYP2C19 expression (37). While CYP2C19 genotype affects VCZ clearance, it does not fully explain age-related exposure differences, and further studies are needed to determine its clinical utility for guiding pediatric dosing. Meropenem may independently influence VCZ exposure by inhibiting CYP450 enzymes, particularly CYP3A4 and CYP2C19, as suggested by both clinical studies in adults and *in vitro* experiments (24, 38), but its effects may be confounded by the inflammatory state (39). In the present study, the combination of meropenem in children aged 2–11 years significantly increased the corrected concentration, if the level of inflammation did not significantly affect VCZ exposure, whereas in the ≥ 12-year-old group, the inhibitory effect of inflammation on CYP2C19 may have masked the drug interaction. PPIs did not affect VCZ C_min_ in this study, contrary to previous reports (23), possibly reflecting ethnic or disease differences. Different PPIs exhibit varying inhibitory potency on key metabolic enzymes, potentially influencing VCZ exposure. In this study, 62.5% of patients concurrently using PPIs received pantoprazole, which has relatively weaker inhibitory effects. This difference may explain the overall lack of consistent PPI-induced changes in VCZ exposure observed in this study. Glucocorticoids significantly reduced VCZ exposure in children ≥12 years of age (40), but not in those 2–11 years, possibly related to a stronger CYP2C19 activity that diminishes their inducing effect (41). The anti-inflammatory effect of glucocorticoids may also influence VCZ metabolism (42), and the exact mechanism needs to be clarified by further studies.

VCZ pharmacokinetics may be influenced by polymorphisms in transporter genes. Previous studies have shown that VCZ may be a substrate for the ABCB1 transporter protein (43) and that ABCB1 rs1045642 significantly affects the steady-state C_min_ of VCZ (36, 44). The present study further demonstrated that VCZ exposure was significantly higher in children aged 2 to <6 years who carried the GA and AA genotypes than the GG genotype; however, this association was not significant in other age groups. Given that some studies have failed to confirm the effect of ABCB1 polymorphisms on VCZ pharmacokinetics (45, 46), and the limited sample size and potential confounding factors in this study, the relationship between ABCB1 rs1045642 and VCZ exposure needs to be elucidated in larger, multicenter cohorts and mechanistic studies.

This study has certain limitations. First, most patients exhibited only mild inflammation, with few showing moderate-to-high CRP levels, which may introduce bias when assessing the relationship between CRP and VCZ exposure. Second, the study did not sufficiently explore other gene polymorphisms, such as FMO3, whose role in pediatric populations remains unclear. Third, due to the difficulty in obtaining pediatric blood samples, the limited sample size precluded further stratified analysis by specific EORTC subtypes. Fourth, excluding concentrations below the LLOQ may introduce slight bias; however, these samples represent less than 10% and are randomly distributed across subgroups, limiting their overall impact. Finally, due to the retrospective design, certain clinical information (e.g., fungal infection type) was incompletely collected. Consequently, these findings require validation through larger prospective studies.

### Study highlights

This study provides robust evidence to support VCZ dose monitoring in pediatric hematology patients. The concentration-efficacy relationship was confirmed through quantitative analysis in the pediatric population. Minimum target plasma concentrations for therapeutic and prophylactic use were identified as 0.41 and 1.115 mg/L, respectively. The results suggest that lower concentrations may be sufficient for prophylaxis, thereby maintaining efficacy while reducing the potential risks associated with prolonged high-dose exposure. In addition, based on age-related physiological development, pharmacokinetic differences and influencing factors were systematically evaluated among children aged 2 to <6, 6 to <12, and ≥12 years. The results indicate that younger children are more prone to inadequate drug exposure, necessitating more precise dosing strategies. Beyond CYP2C19 genotype, gender, inflammatory status, concomitant medications, and transporter gene polymorphisms also significantly influence VCZ exposure across different age groups. Overall, these findings provide a scientific basis for establishing pediatric-specific plasma concentration targets and individualized VCZ dosing and TDM strategies.

## SUPPLEMENTAL MATERIAL

Supplemental Material aac.01169-25-s0001.docx Tables S1 to S4.

ASM does not own the copyrights to Supplemental Material that may be linked to, or accessed through, an article. The authors have granted ASM a non-exclusive, world-wide license to publish the Supplemental Material files. Please contact the corresponding author directly for reuse.
