# Supplementary material for: Optimization strategies for voriconazole dosing in pediatric populations: integrating therapeutic indications and age-stratified pharmacokinetics
Source: Antimicrob Agents Chemother. 2026 Feb 13;70(3):e01169-25. doi: 10.1128/aac.01169-25 (PMC12959138; doi:10.1128/aac.01169-25)
Supplement: Supplemental Material — Tables S1 to S4. [file aac.01169-25-s0001.docx]

**Supplementary Table 1. Baseline of patients in the prevention group.**

| Parameters | Prophylactic therapy No. of patients (%) or median [interquartile range, IQR] | |  |
| --- | --- | --- | --- |
|  | Failure(n=23) | Success (n=80) | *P* value |
| C_min_ | 0.34[0.14,1.28] | 1.29[0.63,2.71] | 0.003 |
| Age (year) | 6.50[4.00,14.00] | 9.00[6.00,12.00] | 0.245 |
| Sex - no. (%) |  |  | 0.773 |
| Male | 14(60.9) | 46(57.5) |  |
| Female | 9(39.1) | 34(42.5) |  |
| BMI (kg/m^2^) | 15.03[14.67,19.06] | 16.44[14.47,17.54] | 0.918 |
| Patient’s underlying condition- no. (%) |  |  | 0.191 |
| AML | 3(13.0) | 21(26.3) |  |
| ALL | 18(78.3) | 43(53.8) |  |
| Other Hematological malignancy | 0(0.0) | 7(8.8) |  |
| Other | 2(8.7) | 9(11.3) |  |

Note: P < 0.05 was considered statistically significant.

**Supplementary Table 2. Baseline of patients in the treatment group.**

| Parameters | Therapeutic therapy No. of patients (%) or median [interquartile range, IQR] | |  |
| --- | --- | --- | --- |
|  | Failure (n=33) | Success (n=96) | *P* value |
| C_min_ | 0.85[0.47,1.72] | 2.15[1.31,3.17] | ＜0.001 |
| Age (year) | 12.00[5.25,15.00] | 10.00[5.00,13.00] | 0.028 |
| Sex - no. (%) |  |  | 0.472 |
| Male | 19(57.6) | 62(64.6) |  |
| Female | 14(42.4) | 34(35.4) |  |
| BMI (kg/m^2^) | 17.15[14.42,20.76] | 15.78[14.50,18.21] | 0.134 |
| Patient’s underlying condition- no. (%) |  |  | 0.760 |
| AML | 19(57.6) | 34(35.4) |  |
| ALL | 11(33.3) | 46(47.9) |  |
| Other Hematological malignancy | 1(3.0) | 7(7.3) |  |
| Other | 2(6.1) | 9(9.4) |  |
| Treatment indication- no. (%) |  |  | 0.896 |
| Targeted | 12(36.4) | 33(34.4) |  |
| Diagnostic-driven | 16(48.5) | 45(46.9) |  |
| Empirical | 5(15.2) | 18(18.8) |  |
| Neutropenia - no. (%)* |  |  | 0.227 |
| Yes | 20(60.6) | 69(66.2) |  |
| No | 13(39.4) | 27(29.8) |  |

Note: * A neutrophil count of fewer than 500 per cubic millimeter at baseline or within the preceding two weeks was considered neutropenia; P < 0.05 was considered statistically significant.

**Supplementary Table 3. The influence of demographic and Pathophysiology variables on CDR of voriconazole among three age groups**

| **Parameter** | **2 to <6 years** | | **6 to <12 years** | | **≥12 years** | |
| --- | --- | --- | --- | --- | --- | --- |
|  | **R** | ***P* value** | **R** | ***P* value** | **R** | ***P* value** |
| Age (year) | 0.052 | 0.604 | 0.123 | 0.171 | 0.026 | 0.776 |
| Weight (kg) | 0.123 | 0.219 | 0.268* | 0.002 | 0.047 | 0.611 |
| BSA (m^2^) | 0.173 | 0.082 | 0.255* | 0.004 | 0.088 | 0.343 |
| CRP (mg/L) | 0.284* | 0.008 | 0.239* | 0.010 | 0.422* | 0.000 |
| Monocytes (10^9^/L) | 0.215* | 0.032 | 0.052 | 0.567 | -0.016 | 0.863 |
| γ-GT (U/L) | 0.324* | 0.001 | 0.148 | 0.104 | 0.182 | 0.054 |
| Urea (mmol/L) | -0.322* | 0.001 | -0.012 | 0.899 | -0.127 | 0.178 |
| Alb (g/L) | -0.161 | 0.116 | -0.041 | 0.652 | -0.15 | 0.113 |
| TP (g/L) | -0.109 | 0.289 | 0.008 | 0.928 | -0.242* | 0.01 |
| ALP (U/L) | -0.037 | 0.721 | 0.031 | 0.733 | 0.216* | 0.021 |

**Note:** * The variable is significant, at the level of 0.05 (double tail).

**Supplementary Table 4. Univariate Analysis of Categorical Variables Affecting Voriconazole CDR Among Three Age Groups**

| **Parameter** | | **2 to <6 years** | | | **6 to <12 years** | | | **≥12 years** | | |
| --- | --- | --- | --- | --- | --- | --- | --- | --- | --- | --- |
|  |  | **N*** | **Median(IQR)** | ***P*** | **N*** | **Median(IQR)** | ***P*** | **N*** | **Median(IQR)** | ***P*** |
| Gender | M | 49 | 0.78(0.28,2.42) | 0.665 | 73 | 1.94(1.10,2.75) | 0.003 | 92 | 1.83(1.11,4.40) | 0.046 |
|  | F | 53 | 0.41(0.24,1.26) |  | 52 | 1.00(0.47,2.58) |  | 27 | 1.31(0.65,2.74) |  |
| CRP (mg/L) | <40 | 81 | 0.49(0.25,1.26) | 0.01 | 101 | 1.63(0.78,2.60) | 0.188 | 87 | 1.53(0.85,2.74) | 0.001 |
|  | ≥40 | 16 | 2.02(0.64,3.19) |  | 14 | 2.48(1.06,2.69) |  | 25 | 4.51(1.85,5.78) |  |
| PPI | N | 86 | 0.51(0.25,1.70) | 0.018 | 105 | 1.63(0.72,2.62) | 0.414 | 91 | 1.77(1.08,4.03) | 0.679 |
|  | Y | 16 | 1.00(0.69,3.57) |  | 20 | 1.88(1.01,2.72) |  | 28 | 1.77(0.83,4.98) |  |
| Carbapenems | N | 68 | 0.44(0.23,1.04) | 0.003 | 79 | 1.24(0.60,2.48) | 0.013 | 62 | 1.61(0.90,2.78) | 0.071 |
|  | Y | 34 | 1.68(0.32,3.16) |  | 46 | 2.18(1.02,3.05) |  | 57 | 2.04(1.21,4.96) |  |
| GC | N | 44 | 0.51(0.26,1.71) | 0.791 | 51 | 1.70(.072,3.00) | 0.494 | 37 | 3.85(1.39,5.48) | 0.002 |
|  | Y | 58 | 0.64(0.25,2.14) |  | 74 | 1.59(0.83,2.58) |  | 80 | 1.61(0.90,2.56) |  |
| *CYP3A5*3* | TT | 24 | 0.85(0.47,2.10) | 0.218 | 51 | 1.83(1.01,2.68) | 0.121 | 38 | 2.82(1.43,5.40) | 0.000 |
|  | CT | 73 | 0.43(0.25,1.68) |  | 66 | 1.60(0.69,2.67) |  | 74 | 1.70(1.06,3.27) |  |
|  | CC | 5 | 2.96(0.10,3.51) |  | 8 | 0.84(0.36,1.15) |  | 7 | 0.37(0.30,0.55) |  |
| *CYP3A4*1G* | CC | 50 | 0.43(0.24,1.22) | 0.11 | 76 | 1.88(0.84,2.79) | 0.141 | 63 | 2.04(1.31,4.81 | 0.005 |
|  | CT | 50 | 0.82(0.31,2.14) |  | 34 | 1.11(0.68,2.61) |  | 52 | 1.54(1.08,2.75) |  |
|  | TT | 2 | 1.84(0.04, -) |  | 15 | 1.58(0.64,2.25) |  | 4 | 0.36(0.31,0.50) |  |
| *CYP3A4* | CC | 84 | 0.59(0.24,1.96) | 0.744 | 104 | 1.68(0.77,2.67) | 0.034 | 93 | 1.83(1.06,4.13) | 0.495 |
|  | CT | 17 | 0.54(0.33,1.85) |  | 15 | 1.68(1.35,2.61) |  | 26 | 1.68(0.73,4.31) |  |
|  | TT | 0 | - |  | 6 | 0.68(0.24,1.00) |  | 0 | - |  |
| *ABCB1* | GG | 50 | 0.32(0.20,0.76) | 0.000 | 42 | 1.49(0.70,2.78) | 0.915 | 36 | 2.10(1.10,4.68) | 0.133 |
|  | GA | 34 | 0.87(0.36,3.02) |  | 65 | 1.65(0.79,2.45) |  | 69 | 1.65(1.01,4.13) |  |
|  | AA | 18 | 2.05(1.03,4.07) |  | 18 | 2.16(0.90,3.14) |  | 14 | 1.61(0.85,2.29) |  |
| *CYP2C19* | NM | 34 | 0.58(0.23,1.14) | <0.001 | 48 | 0.96(0.55,1.54) | <0.001 | 61 | 1.58(0.92,3.07) | 0.075 |
|  | IM | 46 | 0.34(0.21,0.98) |  | 59 | 2.08(1.07,2.08) |  | 46 | 1.92(1.15,4.28) |  |
|  | PM | 21 | 2.88(0.77,3.59) |  | 18 | 2.77(2.58,4.82) |  | 12 | 4.07(1.20,6.95) |  |

**Note**: N*, number; M, male; F, female;N, patients without concomitant medication; Y, patients with concomitant medication; *P<0.05* was considered statically significant.
